# Postural Sway Modulation and Executive Function Performance During the N-Back Task in Younger and Older Adults

**DOI:** 10.1093/geroni/igaf122.3996

**Published:** 2025-12-31

**Authors:** Azizah Jor’dan, Anastasiia Shmidt, Olga Gjika, Ikechukwu Iloputaife, Wanting Yu, Jonathan Bean, Brad Manor

**Affiliations:** University of Massachusetts Boston, Boston, Massachusetts, United States; University of Massachusetts Boston, Boston, Massachusetts, United States; University of Massachusetts Boston, Boston, Massachusetts, United States; Hebrew SeniorLife, Boston, Massachusetts, United States; Hebrew SeniorLife, Boston, Massachusetts, United States; VA Boston Healthcare System, Boston, Massachusetts, United States; Hebrew SeniorLife/Harvard Medical School, Boston, Massachusetts, United States

## Abstract

Maintaining postural control during cognitively demanding “dual tasks” is critical for daily functioning, especially in older adults. Prior work suggests that postural sway is modulated based on the demands of the secondary task to facilitate visual performance. However, it is unclear whether postural sway adapts to facilitate performance on the n-back task of executive function, or whether age influences this relationship. Twenty-three healthy young (26±3 years) and 24 older (76±6 years) adults performed the less demanding identify X (IdX) and more demanding 2-back tasks while standing. Postural sway acceleration (elliptical area, range, and path length) was measured with a wearable motion sensor. N-back task efficiency was reported indexed with the Balance Integration Score (BIS; composite of reaction time and accuracy; lower = better efficiency). During the IdX task, greater sway magnitude was linked to poorer efficiency across participants (p = 0.02), though this attenuated after adjusting for age, sex, and BMI (p = 0.08). In young adults, greater sway magnitude and longer range showed non-significant trends toward worse efficiency (p = 0.07-0.08), trends which were absent in older adults (p > 0.18). In the 2-back task, shorter path length showed non-significant trends toward poorer efficiency both across groups (p = 0.07) and within the older group only (p = 0.08). These results indicate that the association between postural sway and task performance is dependent upon age group and task difficulty. Older adults may not modulate sway to support cognitive efficiency under low demands but may show unique cognitive-motor interactions during more complex tasks.

